# Optometry independent prescribing during COVID lockdown in Wales

**DOI:** 10.1111/opo.13028

**Published:** 2022-08-12

**Authors:** Paul Cottrell, Rachel North, Nik Sheen, Barbara Ryan

**Affiliations:** ^1^ School of Optometry and Vision Sciences Cardiff University Cardiff UK; ^2^ Powys Teaching Health Board Powys UK; ^3^ Health Education & Improvement Wales Nantgarw UK

**Keywords:** COVID‐19, independent prescribing, optometry, self‐care, urgent eye care

## Abstract

**Introduction:**

During the COVID‐19 lockdown, primary care optometry services in Wales moved to a hub model of provision. Three independent prescribing models were available in different areas: a commissioned Independent Prescribing Optometry Service (IPOS), independent prescribers that were not commissioned and no independent prescribers available. This allowed a unique opportunity for comparison.

**Method:**

Optometry practices completed an online survey for each patient episode. Analysis of the data gave insight into patient presentation to urgent eye services and the drugs prescribed by optometrists. Medicines prescribed, sold or given and onward referral were compared between areas with an IPOS service (*n* = 2), those with prescribers but no commissioned service (*n* = 2) and those with no prescribers (*n* = 2).

**Results:**

Data from 22,434 reported patient episodes from 81 optometry practices in six health boards between 14 April 2020 and 30 June 2020 were analysed. Urgent care accounted for 10,997 (49.02%) first appointments and 1777 (7.92%) follow‐ups. Most (18,006, 80.26%) patients self‐referred. The most common presenting symptom was ‘Eye pain/discomfort’ (4818, 43.81% of urgent attendances). Anterior segment pathology was the most reported finding at first (6078, 55.27%) and follow‐up (1316, 74.06%) urgent care appointments. Topical steroids (373, 25.99% of prescriptions) were the most prescribed medications. More medications were prescribed in areas with an IPOS service (1136, 79.16% of prescriptions) than areas with prescribers but no commissioned service. There were more follow‐up appointments in optometric practice and fewer urgent referrals to ophthalmology in IPOS areas.

**Conclusion:**

Urgent care services were most utilised by patients with discomfort caused by anterior eye conditions. IPOS services enabled optometrists to manage conditions to resolution without referral and without reduction in medications sold or given. Commissioners should recognise the value in reducing burden in urgent ophthalmology and the need for follow‐up as part of a commissioned independent prescribing service.

**Key points**

- During the COVID‐19 lockdown, urgent eye care services were most utilised by patients with ocular discomfort caused by anterior eye conditions.
- Areas with commissioned prescribing services saw increased optometrist prescribing and reduced urgent referrals into ophthalmology services.
- When developing future optometry prescribing services, health commissioners should make allowance for on‐going follow‐up as our findings suggest these will be necessary.

## INTRODUCTION

Like other primary care providers, optometry services were significantly affected by the COVID‐19 lockdown and had to adapt quickly to ever changing and unprecedented circumstances. Routine optometric services were stopped in all four UK nations and different models of care were used to ensure on‐going essential and urgent services. Urgent eye care services in primary care optometry had proven safe and effective, and had reduced the volume of patient attendances at hospitals for eye problems in England^1^ and Wales^2^ long before the COVID‐19 pandemic began. Wales moved optometry services to a Primary Care Cluster‐based network of hub practices, which provided urgent and essential care.^3^ Hub practices had agreed with the Welsh Government to be open during the pandemic and were strategically located in every health board and every region in Wales so that there was equity of access for patients in Wales during the COVID‐19 pandemic.

Following support from health boards and government, the number of independent prescribing optometrists in Wales had increased to 45, approximately 6% of the profession, and National Health Service (NHS) prescription pads were available in all health boards. There remain on‐going challenges in the more successful adopter professions for non‐medical independent prescribing.^4^, ^5^, ^6^ Graham‐Clarke et al.^7^ identified that adoption of independent prescribing is more easily achieved in situations where prescribing becomes part of the overall care for the patient. A systematic review by Noblet et al.^8^ described complex multifactorial barriers to independent prescribing, including systems and professional factors, which may explain the small numbers of independent prescribing (IP) optometrists in the United Kingdom.

An ‘Independent Prescribing Optometry Service’ (IPOS) was developed and commissioned in three of the seven health boards in Wales either just prior to or during the first COVID lockdown at the end of March 2020. Health boards commissioned IPOS in primary care optometry practices to ensure that there was independent prescribing cover on each day of the week, but not all practices provided IPOS for all of their opening hours. Local practices without independent prescribing optometrists could refer to an IPOS practice when they expected prescribing would be necessary based on triage or when they discovered a need for prescribing on examination. It was hoped that the use of IPOS in these circumstances would reduce the need for hospital visits for urgent eye care.

Three health boards (Cardiff & Vale University Health Board, Cwm Taf Morgannwg University Health Board and Hywel Dda University Health Board) commissioned an IPOS service. Two health boards (Aneurin Bevan University Health Board and Powys Teaching Health Board) had prescribing optometrists working within the health board but no commissioned IPOS service. Two Health Boards (Betsi Cadwaladr University Health Board and Swansea Bay University Health Board) did not have any prescribing optometrists. Differences in independent prescribing between health boards provided a unique opportunity to compare outcomes. This study aimed to describe optometrist prescribing practices during the COVID‐19 pandemic in Wales, and the differences between areas with a formal IPOS, with prescribing optometrists but no formal IPOS, and without prescribing optometrists.

## METHODS

### Survey design

An online survey was produced through collaboration among the Welsh Government, the Welsh Optometric Committee, Optometry Wales, health board optometric advisers and Cardiff University, and was used to gather data from 14 April to 30 June 2020. All practices that remained open as local eye care hubs during the lockdown period were required to complete the survey for each patient episode that would usually have been paid for by the NHS under either General Ophthalmic Services (GOS) or Eye Health Examination Wales (EHEW). Identical surveys were used in six of the seven health boards in Wales, with the remaining health board using a locally designed survey to capture similar data. Only data captured using the six identical surveys were included in this project. This includes data from Aneurin Bevan University Health Board (ABUHB), Betsi Cadwaladr University Health Board (BCUHB), Cardiff & Vale University Health Board (CVUHB), Cwm Taf Morgannwg University Health Board (CTMUHB), Powys Teaching Health Board (PTHB) and Swansea Bay University Health Board (SBUHB).

Practices inputted the information onto a Jisc online survey (jisc.ac.uk). The online survey was designed to replicate the form used to record audit information and submit financial claims for Eye Health Examination Wales examinations. Information about the patient demographics, appointment types, presenting symptoms, clinical findings and outcomes was captured using a mix of selection lists, multiple choice selections, multiple answer selections and free text fields. Free text entry fields were used to capture data where no selection from a list or multiple choice was appropriate, usually by inclusion of an ‘Other’ selection after which detail was requested in a free text field. All questions were mandatory for completion. Surveys in each of the six areas asked identical questions and had identical answer selections for all questions except for selection of practice name from a selection list. In this case, the list was tailored to each health board, with only practices within the health board listed for selection.

There is no patient identifiable information in the surveys. Ethical approval for review and analysis of this data was provided by the Aneurin Bevan University Health Board Research & Development Department and the Cardiff University School of Optometry & Vision Sciences Research Ethics Committee (Ref 1550).

The complete survey can be found in Appendix 1.

### Data retrieval

For this analysis the raw data sheet for each survey was downloaded from Jisc online surveys for the period from 14 April 2020 to 30 June 2020. The raw, uncoded data for each survey were downloaded in Microsoft Excel 2007 and later (.xlsx) format.

### Data analysis

The data were analysed using Microsoft Excel for Microsoft 365 (Microsoft.com) and IBM SPSS Statistics 26 (ibm.com). Descriptive statistics were used to describe the demographics of patients attending optometry services.

The drugs prescribed were allocated to an appropriate drug classification group (e.g., topical or systemic antibiotic). The drugs were further grouped by administration method, either systemic or topical, and totals for each of these groups found by adding each relevant drug totals.

Data from health boards were split into three groups by prescribing service: those with an IPOS (*n* = 2), those with prescribing optometrists in the area but no commissioned IPOS (*n* = 2) and those with no prescribing (*n* = 2). The one remaining health board in West Wales had an IPOS service but was not included as they used a different survey. Health boards were further grouped into ‘prescribing group’, either ‘IPOS’, those with a formally commissioned prescribing service (*n* = 2) and ‘non IPOS’, those without a formally commissioned prescribing service (*n* = 4) before Chi square tests were performed to identify association of each of the outcomes with either ‘IPOS’ or ‘non IPOS’ group. The total prescriptions for each group were compared using Chi square test.

The total of each prescribing outcome (‘Referral to General Medical Practitioner (GP) to prescribe’, ‘Referral Ophthalmologist to prescribe’, ‘Medication prescription issued’, ‘Referral to pharmacist for medication’, ‘Sell/give medication’) in each health board, and percentage of all appointments for each group were calculated to allow comparison.

As this was a post‐hoc analysis, to avoid Type 1 errors, *p* < 0.05 would be adjusted to <0.004 as significant with Bonferroni correction. Hence, *p* < 0.004 was used as significant in the analysis of this large dataset. Where free text fields had been used, answers were variable, not enabling analysis.

## RESULTS

### Patient demographics

A total of 81 practices across the research area conducted 22,434 interactions during the period 14 April 2020 to 30 June 2020. Twelve thousand seven hundred and sixty‐eight (56.91%) interactions were with female patients and 9661 (43.06%) were with male patients. Five patients had gender listed as ‘Other’. When grouped by decade, female patients exceeded male patients in all age groups except the under 10s. The greatest number of encounters was in the 61–70 age group (3378, 15.06%).

### Patient appointment types

Of the 22,434 interactions across the research area, 10,997 (49.02%) interactions were for urgent eye care, with an additional 1777 (7.92%) associated follow‐ups, 4540 (20.24%) interactions would have been considered part of the GOS in times outside COVID‐19, and the remaining 5120 (22.82%) interactions were classified as ‘Other’, for example post‐cataract services and spectacle intolerance rechecks. The highest specific proportion of appointments were for urgent care in five health boards, and there were more ‘Other’ interactions than urgent care attendances in ABUHB.

### Referrals into optometry services

A large majority (18,006, 80.26%) of patients self‐referred into the service. In five health boards, the highest proportion of referrals into the service from other professionals came from GPs; in CVUHB, an IPOS area, most referrals were received from other optometrists (Table 1).

**TABLE 1:** Referral source for all patient interactions in optometry services during the COVID‐19 lockdown

	Total		Aneurin Bevan University Health Board		Betsi Cadwaladr University Health Board		Cardiff & Vale University Health Board		Cwm Taf Morgannwg University Health Board		Powys Teaching Health Board		Swansea Bay University Health Board	
Total	22,434		6254		3250		4376		4822		844		2888	
Self‐referred	**18,006**	**(80.26%)**	**5400**	**(86.34%)**	**2424**	**(74.58%)**	**3222**	**(73.63%)**	**3974**	**(82.41%)**	**670**	**(79.38%)**	**2316**	**(80.19%)**
General Medical Practitioner	1966	(8.76%)	434	(6.94%)	466	(14.34%)	351	(8.02%)	375	(7.78%)	101	(11.97%)	239	(8.28%)
Optometrist	1085	(4.84%)	237	(3.79%)	86	(2.65%)	479	(10.95%)	208	(4.31%)	6	(0.71%)	69	(2.39%)
Ophthalmologist	519	(2.31%)	52	(0.83%)	77	(2.37%)	138	(3.15%)	130	(2.70%)	10	(1.18%)	112	(3.88%)
Pharmacist	211	(0.94%)	49	(0.78%)	60	(1.85%)	48	(1.10%)	27	(0.56%)	13	(1.54%)	14	(0.48%)
Other HES professional	189	(0.84%)	21	(0.34%)	58	(1.78%)	44	(1.01%)	35	(0.73%)	17	(2.01%)	14	(0.48%)
Other	458	(2.04%)	61	(0.98%)	79	(2.43%)	94	(2.15%)	73	(1.51%)	27	(3.20%)	124	(4.29%)

*Note*: Bold = group with highest proportion of patients.

### Presenting symptoms

Reporting practitioners were able to make multiple selections to record all symptoms reported by the patient. The most common presenting symptom in the study area was ‘Eye pain/discomfort’, reported at 4818 (43.81%) urgent care attendances. Red eye (2935, 26.69%) and acute vision problems (2460, 22.37%) were second and third, respectively. The same order of frequency was found in all health boards except BCUHB and SBUHB, where more acute vision problems (BCUHB 568, 24.11%; SBUHB 310, 23.31%) were reported than red eye symptoms (BCU 563, 23.90%; SBUHB 305, 22.93%).

The proportion of patients reporting red eye or pain increased significantly between the first and follow‐up appointments. Other symptoms showed either no significant change or a reduction in the proportion of appointments (Table 2).

**TABLE 2:** Change in symptom presentation between assessment and follow‐up urgent care visits

	Assessment (%)	Follow up (%)	Change (%)	*Χ* ^2^(2), *p*
None	0.84	12.55	11.71	872.580, <0.001
Acute vision problem	22.37	13.22	−9.15	72.860, <0.001
Chronic vision problem	5.85	5.97	0.12	0.039, 0.84
Red eye	26.69	34.61	**7.92**	**47.816, <0.001**
Flashes	6.85	1.97	−4.88	62.881, <0.001
Floaters	12.54	3.83	−8.71	115.625, <0.001
Eye pain/discomfort	43.81	55.49	**11.67**	**84.106, <0.001**
Headaches	11.53	2.14	−9.39	147.019, <0.001
Diplopia	1.58	0.62	−0.96	9.945, 0.002
Other	8.07	6.58	−1.48	4.638, 0.03

*Note*: Bold = significant positive change between first and follow‐up appointments; multiple selections could be made hence totals exceed 100%.

### Examination findings

Reporting practitioners were asked to select all clinical findings and hence were able to make multiple selections for each patient episode. Anterior segment findings were present in 6078 (55.27%) urgent care assessment attendances. The most common findings across the study area were ‘Eyelid/ Eyelash/ Lacrimal’ (1607, 14.61%), ‘Conjunctiva’ (1595, 14.50%), ‘No Clinical Abnormality’ (1558, 14.17%) and ‘Dry Eye/Meibomian Gland Dysfunction’ (1554, 14.13%).

For follow‐up appointments, ‘Anterior Segment’ findings accounted for a larger proportion of the cases (1316, 74.06%).

The greatest increase overall between first and follow‐up visits was in ‘Cornea/Sclera’ findings followed by ‘Iris/Ciliary Body’, whilst the greatest decrease was in ‘Other’, followed by ‘Posterior Vitreous Detachment/Other vitreous’ (Table 3).

**TABLE 3:** Change in clinical findings between first and follow‐up urgent care visits

	First (%)	Follow up (%)	Change (%)	*Χ* ^2^(2), *p*
No clinical abnormality	14.17	13.11	−1.06	1.414, 0.23
Dry eye/Meibomian gland dysfunction	14.13	14.97	0.84	0.879, 0.35
Eyelid/Eyelash/Lacrimal/Orbit	14.61	15.08	0.47	0.268, 0.60
Foreign Body/Other trauma	4.87	4.28	−0.60	1.196, 0.27
Conjunctiva	14.50	11.99	−2.52	7.979, 0.005
Cornea/Sclera	10.47	26.00	**15.53**	**334.524, <0.001**
Cataract/Lens/Intraocular Lens/Posterior capsule opacification	3.35	1.86	−1.49	11.159, 0.001
Iris/Ciliary body	3.07	12.44	**9.36**	**320.498, <0.001**
Dry Age‐related macular degeneration	1.54	0.51	−1.03	11.819, 0.001
Wet Age‐related macular degeneration	1.59	0.23	−1.37	20.668, <0.001
Other macula	1.52	1.24	−0.28	0.826, 0.36
Retinal break/detachment	1.45	0.45	−1.00	11.754, 0.001
Other retinal	2.20	1.01	−1.19	10.822, 0.001
Posterior vitreous detachment/Other vitreous	7.66	2.31	−5.35	68.032, <0.001
Suspect glaucoma/Ocular hypertension	0.56	0.84	0.28	2.006, 0.16
Optic nerve/Visual pathway/Migraine	4.98	1.29	−3.69	48.750, <0.001
Ocular motor balance/refractive error adults	1.87	0.96	−0.92	7.493, 0.006
Ocular motor balance/refractive error children	0.65	0.11	−0.53	7.651, 0.006
Other	10.61	5.35	−5.27	47.646, <0.001

*Note*: Bold = significant positive change; multiple selections could be made hence totals exceed 100%.

### Outcomes

Practitioners were able to record multiple answers for outcome. The most common outcome from urgent care appointments was ‘Advice/Regular Review’, reported in 7246 (65.89%) appointments overall.

Recall rate for follow‐up varied between health boards. CVUHB had the highest rate of recall for follow‐up appointments, where one follow‐up was arranged for every 3.84 urgent care attendances. BCUHB had the lowest follow‐up ratio with one follow‐up arranged for every 13.24 urgent care attendances.

At follow‐up appointments, the rate of ‘Follow‐up arranged’ increased. Across the research area for each 100 patients who attended for urgent care, 12 received a follow‐up, of whom two required a second follow‐up. The highest follow‐up rates were identified in CVUHB, where of 100 urgent care attendances, 19 would receive a follow‐up appointment, around five would require a second follow‐up. Referrals to other professionals were significantly lower at follow‐up visits than initial visits (Table 4).

**TABLE 4:** Change in outcomes between first and follow‐up urgent care

	First (%)	Follow up (%)	Change (%)	*Χ* ^2^(2), *p*
Advice/regular review	65.89	65.45	−0.44	0.134, 0.72
Follow‐up	12.19	16.49	4.30	25.409, <0.001
Prescribing outcome	31.35	30.95	−0.40	0.116, 0.73
Referral outcome	36.12	23.52	**−13.40**	**107.609, <0.001**
Foreign body/Eyelash removal	2.43	1.46	−0.96	6.354, 0.01
Spectacle prescription issued	4.13	2.48	−1.65	11.148, 0.001
Spectacles made up	4.16	2.76	−1.41	7.949, 0.005
Spectacle repair	0.05	0.06	0.00	0.001, 0.98
Low vision aid repair/replacement	0.05	0.06	0.00	0.001, 0.98
Report to general medical practitioner	28.61	22.96	−5.65	24.299, <0.001
Other	10.42	10.35	−0.07	0.007, 0.93

*Note*: Bold = notable finding; multiple selections could be made hence totals exceed 100%.

### Medications prescribed

**FIGURE 1:**
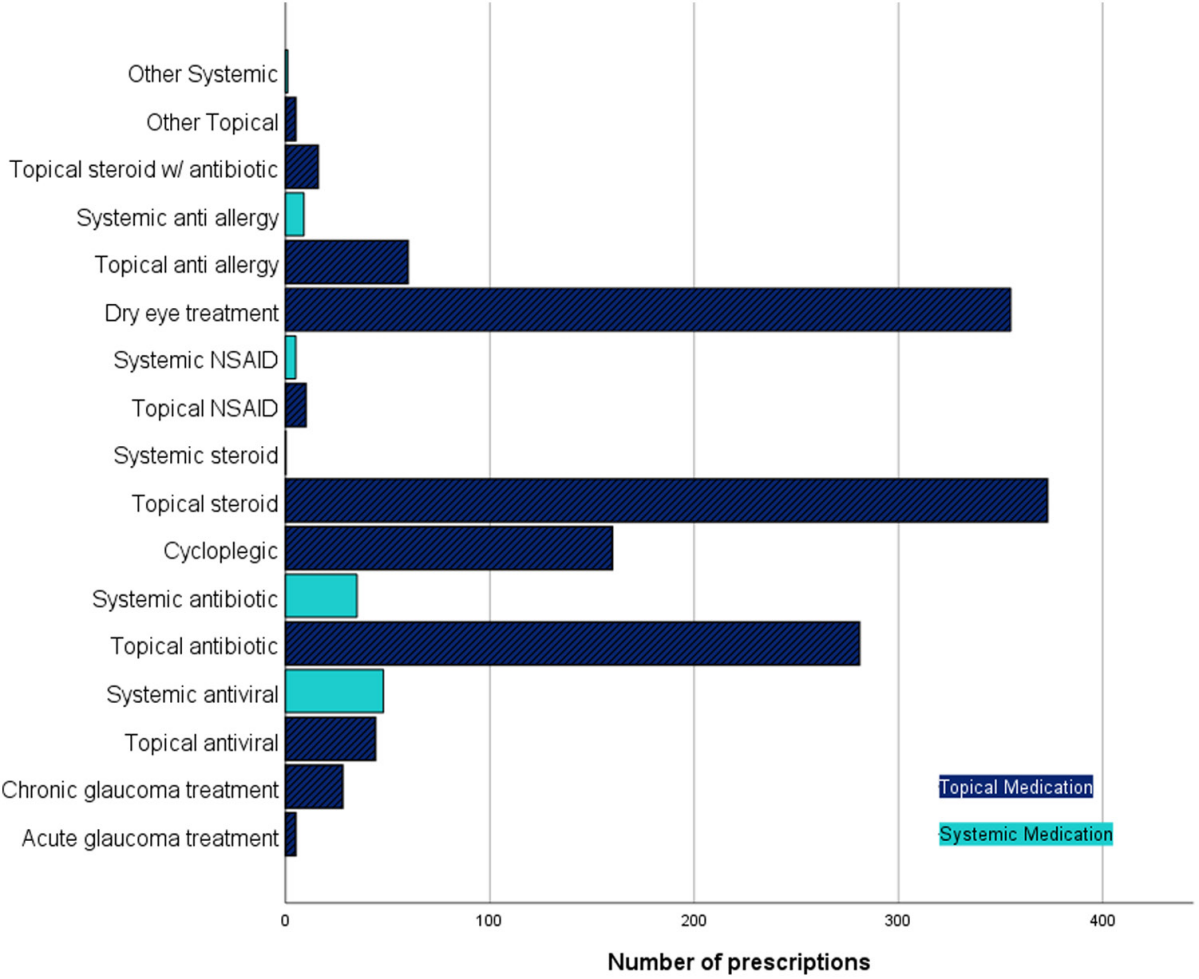
Medications prescribed by optometrists during the COVID‐19 lockdown. NSAID, Non‐steroidal anti‐inflammatory drug.

During the COVID‐19 pandemic, optometrists in the six health boards considered in this analysis prescribed a total of 1435 medications, of which 1332 (92.82%) were topical ocular treatments and 103 (7.18%) were systemic medications. The most prescribed group of medications were topical steroids (373 prescriptions, 25.99%), followed by dry eye treatments (355, 24.74%), topical antibiotics (281, 19.58%) and cycloplegics (160, 11.15%). All other medication groups accounted for less than 5% of prescriptions issued (Figure 1).

Of the systemic medication prescriptions, antivirals were most prescribed, accounting for 3.34% (48 prescriptions) of the total prescriptions but 41.74% of the systemic medication prescriptions.

### The effect of commissioned IPOSs

When health boards were grouped by prescribing service there was a statistically significant association between prescriptions issued and prescribing group (*Χ* ^2^(2) = 1005.73, *p* < 0.001). Of the total 1435 medication prescriptions, 1136 (79.16%) were issued in health boards with IPOS services, 288 (20.07%) in health boards with prescribers but no commissioned service and 11 (0.77%) were recorded in areas with no prescribers.

**FIGURE 2:**
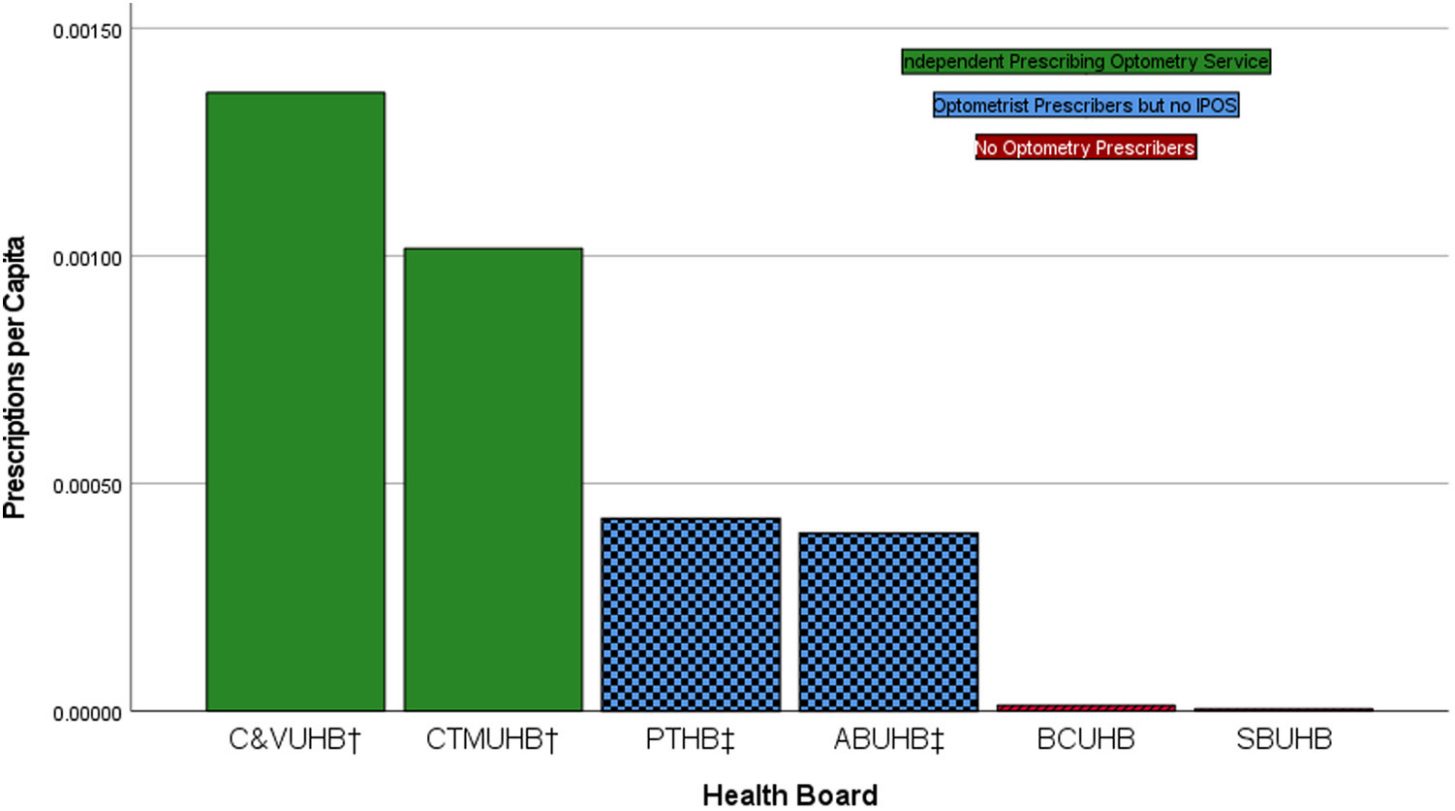
Medication prescriptions per capita by health board. ^†^Health boards with commissioned IPOS; ^‡^Health boards with prescribers but not commissioned independent prescribing optometry services (IPOS). ABUHB, Aneurin Bevan University Health Board; BCUHB, Betsi Cadwaladr University Health Board; C&VUHB, Cardiff & Vale University Health Board; CTMUHB, Cwm Taf Morgannwg University Health Board; PTHB, Powys Teaching Health Board; SUHB, Swansea Bay University Health Board.

When corrected for population, combined areas with IPOS services provided 0.00120 prescriptions per capita during the lockdown period, three times the relative prescriptions in areas with optometry prescribers but no IPOS (0.00040/capita) (Figure 2).

### Prescribing outcomes including referral for prescribing

Of the 22,434 appointments during the lockdown period, 4354 (19.41%) led to prescribing outcomes. The IPOS areas ranked first and third in likelihood of prescribing outcome, either by medication prescribed by an optometrist or referral onto another professional for prescribing. A prescribing outcome was most likely in CVUHB (1117 of 4376 appointments, 25.53%) and third most likely in CTMUHB (1025 of 4822, 21.26%). BCUHB, an area with no prescribers, showed more referrals to a GP for prescribing than all other health boards (163 of 3250, 5.02%). The two health boards with no prescribers ranked first and third in the proportion of appointments ending with a referral to a pharmacy for medication (BCUHB 274 of 3250, 8.43%; SBUHB 179 of 2888, 6.20%).

There were associations between prescribing group and reduced referrals to GP for prescribing (*Χ* ^2^(2) = 15.712, *p* < 0.001) and increased referrals to a pharmacy for prescribing (*Χ* ^2^(2) = 26.542, *p* < 0.001). No association was found between prescribing group and referral to ophthalmology for prescribing, for which the volume of referrals was very low in all health boards, although highest in the two areas with no prescribers (BCUHB 10 of 3250, 0.31%; SBUHB 13 of 2888, 0.45%) (Figure 4).

### Selling and giving medication

**FIGURE 3:**
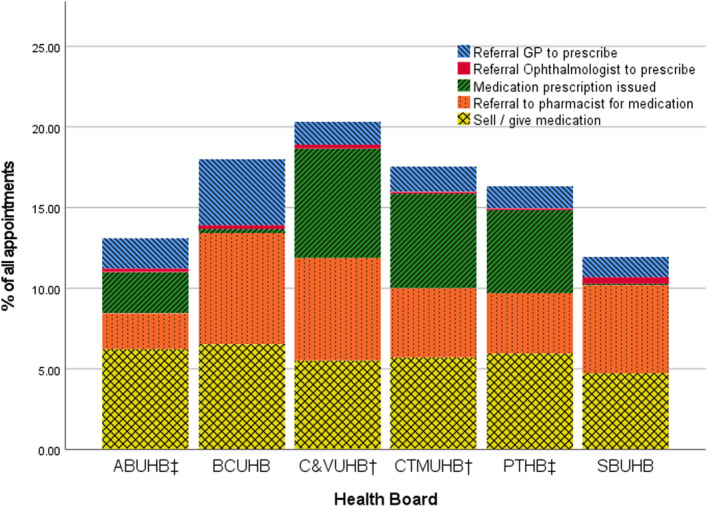
Appointments with prescribing outcomes by health board. ABUHB, Aneurin Bevan University Health Board; BCUHB, Betsi Cadwaladr University Health Board; C&VUHB, Cardiff & Vale University Health Board; CTMUHB, Cwm Taf Morgannwg University Health Board; GP, General medical practitioner; IPOS, Independent prescribing optometry services; PTHB, Powys Teaching Health Board; SBUHB, Swansea Bay University Health Board; ^†^Health Boards with commissioned IPOS; ^‡^Health Boards with prescribers but not commissioned IPOS.

Optometrists managing acute or minor eye conditions may give medication to a patient or advise them to buy medications over the counter. Across the research area, 6.94% (1558 of 22,434) of appointments led to a ‘Sell/give medication’ outcome. There was no association between the proportion of appointments with the outcome ‘sell/give medication’ and prescribing group (*Χ* ^2^(2) = 0.944, *p* = 0.62). ‘Sell/give medication’ is most likely the option selected when the optometry practice advises self‐care rather than actively prescribing for the patient. When all prescribing outcomes are assessed across the research area, self‐care was the most likely prescribing outcome (1558 of 4354 prescribing outcomes, 35.78%), with referral to pharmacy the next most likely prescribing outcome (1251, 28.73%) (Figure 3). When only the four health boards with independent prescribing optometrists were considered, self‐care remained the most likely prescribing outcome (1145 of 3249 prescribing outcomes, 35.24%) but medication prescription issued by an optometrist was the second most likely prescribing outcome (951, 29.27%).

### Onward referral

**FIGURE 4:**
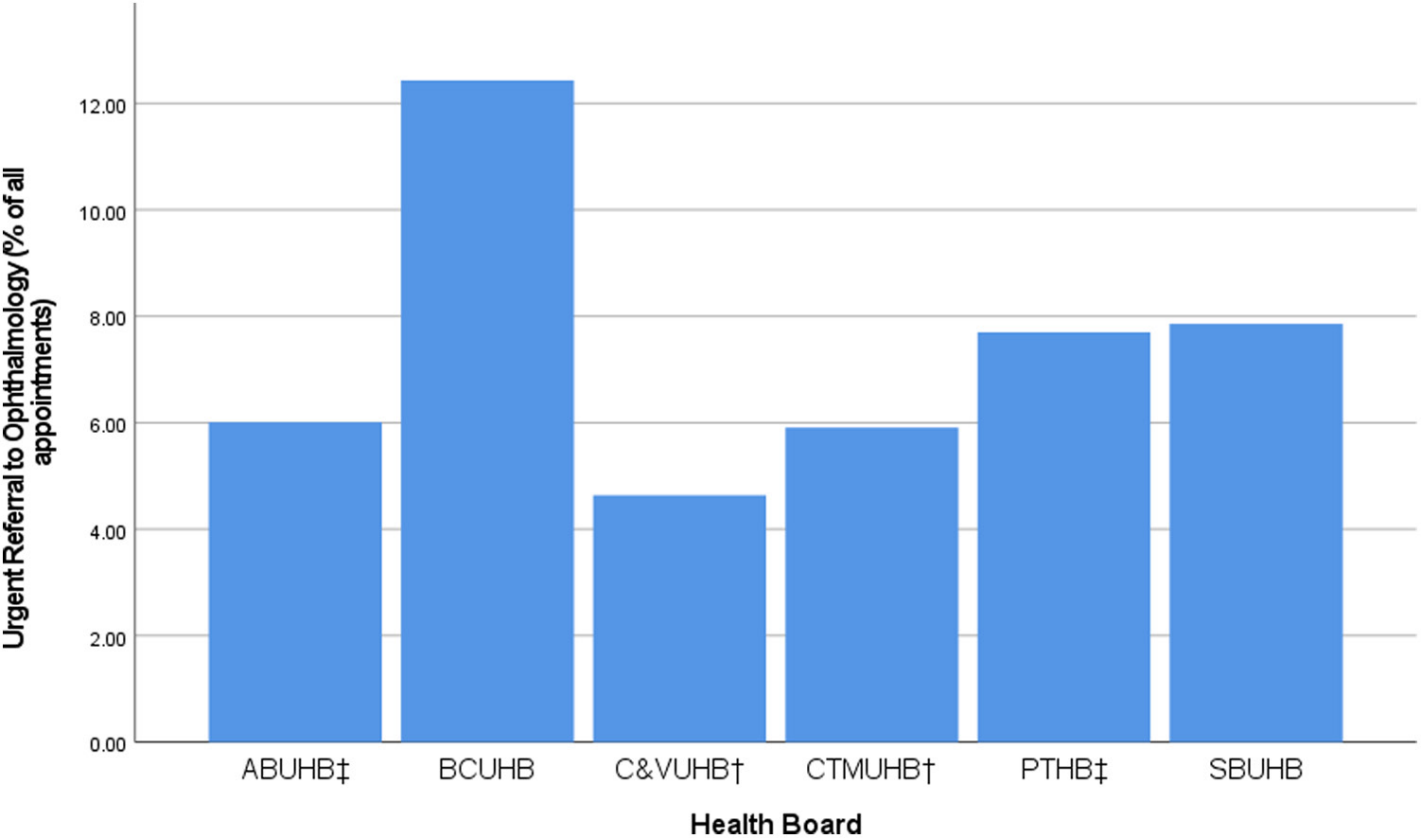
Proportion of appointments with urgent referral to ophthalmology outcome in each health board. ABUHB, Aneurin Bevan University Health Board; BCUHB, Betsi Cadwaladr University Health Board; C&VUHB, Cardiff & Vale University Health Board; CTMUHB, Cwm Taf Morgannwg University Health Board; IPOS, independent prescribing optometry services; PTHB, Powys Teaching Health Board; SBUHB, Swansea Bay University Health Board. ^†^Health Boards with commissioned IPOS; ^‡^Health Boards with prescribers but not commissioned IPOS.

There were 4929 (21.97% of 22,434) referrals following optometry consultations. A total of 2071 (9.23%) appointments ended in a referral to ophthalmology, 1300 (5.79%) to GPs, 1251 (5.58%) to pharmacies and 307 (1.37%) to other professionals. Health boards with IPOS service saw the fewest total referrals to ophthalmology (CVUHB 321, 7.34%; CTMUHB 361, 7.49%) and fewest urgent referrals to ophthalmology (CVUHB 203, 4.64%; CTMUHB 285, 5.91%) (Figure 4). The two health boards with no prescribing saw the highest proportion of referrals for urgent ophthalmology assessment (BCUHB 404, 12.43%; SBUHB 227, 7.86%), although the reason for BCUHB experiencing markedly higher urgent referral rates than all other health boards including SBUHB is unclear.

Independent Prescribing Optometry Service areas rank first and third in ‘Referral to Other Professional’ category, likely indicating referral to optometry practices for IPOS. The highest rate of referral in all categories except ‘Referral to Other Professional’ was in BCUHB, an area without any prescribing optometrists. Significant association was found between the prescribing group and referral rates for urgent ophthalmology referrals (*Χ* ^2^(2) = 65.458, *p* < 0.001), and referrals to GP (*Χ* ^2^(2) = 11.357, *p* = 0.001), with a higher proportion of referrals made in non IPOS areas. A significant association was found in referral to pharmacy (*Χ* ^2^(2) = 26.542, *p* < 0.001) and referral to other professionals (*Χ* ^2^(2) = 26.586, *p* < 0.001), with higher referral rates in IPOS areas. There was no association between routine referral to ophthalmology and prescribing group (*Χ* ^2^(2) = 1.992, *p* = 0.16).

## DISCUSSION

This work reflects a unique opportunity to learn from a large sample of patient episodes representing all patient contacts from open practices during a 10‐week period in six health boards across Wales. This time saw rapid service development including the implementation of a commissioned IPOS.

During the COVID‐19 lockdown period, most consultations were sought for urgent eye care. This likely reflects legislation which restricted public movement and guidance to the optometry profession to cease routine eye care. As COVID‐19 was accelerating in the United Kingdom, the Welsh Government passed legislation that required people not to leave their local area without good reason.^9^ Seeking health care was considered an acceptable reason to leave home, although patients were encouraged to stay close to home.

### Management by optometry services

Of the 22,434 patient interactions during the research period, 17,505 (78.03%) were managed completely by optometry without any onward referral.

The data show that 9.23% of all patients during the COVID‐19 lockdown were referred to ophthalmology and 5.79% to their GP; both significantly lower than the previous Welsh Eye Care Service findings by McAlinden et al.^10^ who reported that 17.6% of patients were referred to ophthalmology and 8.6% to their GP following optometry review.

### Independent prescribing optometry services

This study benefitted from having six health board areas, two of which commissioned an IPOS, two had prescribing optometrists but no commissioned service and two had no prescribing optometrists. Prescribing appeared to be very low but not zero in areas without optometry prescribers, suggesting a small amount of error in completion of the online surveys. Prescribing rates were significantly higher in areas with a commissioned prescribing service. The result of this was a reduction in referrals to ophthalmology for urgent eye care from optometrists, suggesting that a formal commissioned IPOS in all areas would reduce the burden on hospital eye services.

Optometrists with appropriate training have been able to independently prescribe since 2008,^11^ and the first prescription pad was issued to a primary care optometrist in Wales in 2018, but in general uptake in the United Kingdom as a whole has been poor.^12^ Noblet et al.^8^ describe a plethora of barriers in the establishment of independent prescribing for non‐medical professionals. The emergence of the COVID‐19 pandemic accelerated the implementation of commissioned services for optometrists who had already taken the decision to train as independent prescribers. Partial government funding for training in Wales has removed a key barrier to entry into independent prescribing, although there remain significant challenges to qualified independent prescribing optometrists in areas without a commissioned IPOS. This is reflected in the difference between prescribing rates per capita in the IPOS and non IPOS health boards.

A commissioned IPOS appears to overcome many barriers outlined by Noblet et al.,^8^ embedding robust governance processes and appropriate remuneration for the service. Good relationships and collaborative working have been highlighted as important factors in success of non‐medical prescribing services.^8^, ^13^ IPOS was developed collaboratively between ophthalmology departments and local optometry leaders in the first commissioning health boards. Harper et al.^14^ noted that the success of a COVID Urgent Eyecare Service (CUES) in Manchester relied on a critical mass of independent prescribing optometrists, while Noblet et al.^8^ cited the importance of long‐term viability and the need for a significant number of independent prescribers in a service. IPOS during COVID‐19 was commissioned to ensure prescribing covers every day of the working week by using different practices, hence providing a robust service.

The IPOS led to increased prescribing, a reduction in optometry referrals for urgent ophthalmology care and to GPs, and did not affect other means of treating minor eye conditions such as selling over the counter drugs or giving these to the patient in an emergency. There was no association between the proportion of patients referred routinely to hospital ophthalmology departments and IPOS availability. This is unsurprising as routine referral is often for confirmation of diagnosis and treatment of long‐term conditions that cannot be managed independently by optometrists.^15^

A higher proportion of referrals to pharmacy in IPOS areas than non IPOS areas is an unexpected finding, and may be due to selection of ‘Refer to Pharmacy’ in the data capture tool to indicate that the patient was given a prescription to take to the pharmacy for medication provision. Further interrogation of the data would be required to establish other possible causes.

Reduction in optometry urgent referrals and referrals to GP compared with previous data suggests that optometrists were able to manage more patients in their practice and refer less, possibly due to a combination of factors including IPOS and a hesitancy for hospitals to accept referrals during the lockdown period. A reduction in the proportion of appointments ending in referral is particularly heartening in a period when care was provided only for urgent and essential cases as triaged by the practice, and during a time when patients were reticent to leave home. It could reasonably be expected for referrals to increase during this time, as patients attended only with specific cause for concern. These findings indicate that optometry practices are now comfortable managing more cases in primary care than was the case when previous comparable research was undertaken by McAlinden et al.^10^ The design of this study did not allow for consideration of false negative referrals which have been found to be a cause for concern in other areas during the COVID‐19 pandemic.^16^ Further research into the safety of optometry services in Wales during the COVID‐19 pandemic would be beneficial.

There were 307 (1.37%) referrals to ‘Other Professionals’. A significantly greater proportion of patients referred to ‘Other Professional’ was observed where a prescribing service was available, likely referrals to optometrists within IPOS. There is an absence of literature regarding inter practice referral within optometry in the United Kingdom despite a growing trend in establishment of enhanced referral refinement services which require inter practice referral.^17^, ^18^ These findings are indicative of som`e willingness of optometrists to refer patients to another practice for care where it is clinically appropriate and necessary, although it must be recognised that optometry practices were working in a very different way to normal during the COVID‐19 lockdown period. There are undeniable barriers to inter‐practice referral in optometry, largely caused by the loss leading and spectacle subsidy aspects of the challenging business model.^19^ Future review of the commissioned IPOS will provide valuable data about optometrists' willingness to refer within the profession rather than to hospital services under normal working circumstances.

### Selling and giving medication

Most of the ‘sell and give’ category will have been patients with minor eye conditions buying medications over the counter in the optometry practice or elsewhere, at the recommendation of the optometrist following a consultation. In a small number of cases, optometrists managing acute eye conditions may give medication to a patient. For example, if they removed a foreign body from the patients' cornea they may give topical antibiotic treatment as a prophylactic until the cornea heals. The data show that medications given to the patient or sold over the counter is the most likely prescribing outcome, accounting for 1558 of 4354 (35.78%) instances.

In a chronic and/or recurring minor eye condition, this could be viewed as promotion of self‐care. The World Health Organisation^20^ defines self‐care as ‘the ability of individuals, families and communities to promote health, prevent disease, maintain health, and to cope with illness and disability with or without the support of a healthcare provider’ whilst the Self Care Forum^21^ considers self‐care as ‘the actions that individuals take for themselves, on behalf of and with others in order to develop, protect, maintain and improve their health, wellbeing or wellness.’ There are significant long‐term benefits to the NHS when patients engage in self‐care rather than seek medical advice. In 2007, it was estimated that £2 billion of NHS resources were spent annually on GP management of minor ailments, with £371 million of the total spent on prescriptions, for which a similar product could be purchased over the counter by the patient.^22^ For minor ocular conditions, this may include dry eye treatments such as artificial tear drops, allergy treatments and chloramphenicol for bacterial conjunctivitis.

In this study, there was no significant difference in the proportion of ‘Sell/give medication’ outcomes between health boards that had a commissioned IPOS, prescribers but no commissioned service or no prescribers. This suggests that whilst optometrists with a prescribing qualification could prescribe medications, they are still advising self‐care appropriately and thus not increasing the burden of prescription costs for the NHS.

### Medications prescribed

This is the first review of medication types prescribed by independent prescribing optometrists in primary care. The medications most prescribed by optometrists were topical steroids (373 prescriptions, 25.99%), dry eye treatments (355, 24.74%) and topical antibiotics (281, 19.58%). It is perhaps unsurprising that optometrists prescribed predominantly topical medications as the most common ocular conditions are treated topically rather than systemically,^23^ and optometrist prescribers are required to work within their usual area of competence.^24^ The profile of medications prescribed by optometrists corresponds well with the presenting symptoms and recorded findings.

### Conditions managed by optometry services

The presenting symptoms and findings recorded at urgent care appointments indicated that patients were more likely to attend due to discomfort than vision change. This fits with recorded findings that most conditions managed were anterior eye problems.

Anterior segment conditions were the most common findings during the COVID‐19 lockdown, accounting for 6078 (55.27%) first attendances and 1316 (74.06%) follow‐ups. The most reported presenting symptoms were ‘Eye pain/discomfort’, ‘Red Eye’ and ‘Acute Vision Problem’. The National Institute for Health and Care Excellence (NICE)^25^ list the serious and sight threatening causes of a red eye as acute glaucoma, corneal ulcer, anterior uveitis, scleritis, trauma, chemical injury and conjunctivitis, the more severe of which are anterior eye conditions that also present with eye pain and acute vision change and require medical management. The UK College of Optometrists^23^ guidelines for treatment of many acute anterior eye conditions includes topical antibiotics and steroids, which were the most and third most prescribed medication groups in these data, respectively.

A significant increase in the percentage of ‘Eye pain / discomfort’ and ‘Red eye’ between the first and follow‐up visits suggests that anterior eye conditions such as uveitis were medically managed by optometrists in practice and followed up appropriately until the condition resolved, with an increase in ‘None’ reported symptoms at follow‐up indicating successful management to resolution. A marked increase in findings of ‘Iris/Ciliary body’ and ‘Cornea/Sclera’ in follow‐up appointments strengthens the consideration that anterior eye conditions such as iritis, corneal defects or corneal ulcers were managed in primary care without need for referral. Significant association with fewer reports at follow‐up appointments include ‘Headache’, ‘Acute Vision Problem’, ‘Flashes’ and ‘Floaters’ symptoms, and ‘Conjunctiva’ and ‘Cataract/Lens/IOL/PCO’ clinical findings, each of which would usually be managed as single attendances in optometric practice, either by giving advice to the patient or referral with the appropriate urgency.

### On‐going follow‐up

During the lockdown period, 297 (16.49%) follow‐up appointments led to further follow‐up, with 239 (81.57%) of these second or further follow‐ups occurring in the two health boards with IPOS. Whilst this is not a surprising finding due to the on‐going nature of the ocular conditions that are medically managed, it is of interest for future service design. Any future services which allow independent prescribing optometrists to use their qualification need to recognise that on‐going follow‐up is required and must make allowances for this.

### Strengths of this study

The situation created during the time of the COVID‐19 lockdown gave an ideal opportunity to learn about how and why people access urgent eye care services. The greatest strengths of this work are the large number of patient episodes recorded, and that all episodes were to be recorded so the sample is likely very close to 100% of the true activity.

### Limitations of this study

This study may be compromised by lack of control over the data collected. Data were input directly into the survey for more than 22,000 episodes by 81 optometry practices during busy clinical days. With such a wide variety of individuals entering data and a large volume of records, variation in the quality of data is unsurprising and echoes previous findings. Following a review of the literature, Fowles and Weiner^26^ observed the quality of data entered into electronic records by clinicians to be questionable, even when the same record system was used by different clinicians within the same organisation.

The survey was built quickly in order to react to the fast‐moving situation early in the COVID‐19 pandemic, and communications that were sent to practices may not have been as clear as they would have been with more time to plan. Some practices and some areas interpreted the instructions differently. This resulted in, for example a significant amount of inappropriate data being inputted as ‘other’ appointment types at the ABUHB. On closer examination, it appears that many of the entries in this circumstance are for patient encounters that would not usually be considered a ‘consultation’. The same issues may be true to a lesser extent in the other health boards.

Whilst the ‘Other’ free text fields were useful in identifying where inappropriate episodes had been inputted, for example where private contact lens consultations had been recorded, their completion was variable and entries not consistent enough to allow proper analysis. The high proportion of ‘Other’ entries across the survey limits analysis.

Errors have been made in data entry for the prescribing questions on the survey. Analysis shows a small number of prescriptions issued in health boards where there were no independent prescribing optometrists. As prescription pads were issued by health boards to practices to ensure that governance processes were adequate, it is impossible for the prescribing optometrists to have moved to a practice in another health board and continued to prescribe. We can be sure that these entries are erroneous; however, the number of erroneous entries is very small. Also, date of appointment was confused for date of birth in some cases. It is likely that the errant date records were evenly spread throughout the data, and so it is unlikely to have affected the result.

## CONCLUSIONS

This work highlights several important findings that are of interest in future service developments. The data show that urgent care services were most utilised by patients with ocular discomfort caused by anterior eye conditions which, particularly with the engagement of optometrist independent prescribers, can be managed to resolution in primary care. Areas where a commissioned prescribing service was used saw increased optometrist prescribing and reduced urgent referrals into ophthalmology services, suggesting that a formal commissioned IPOS in all areas would reduce the burden on hospital eye services. The presence of commissioned optometry prescribing services had no effect on the proportion of patients receiving self‐care advice from optometrists, showing that prescribing optometry services do not adversely affect NHS spending on prescriptions when over‐the‐counter sales are available. As more funded IPOS are established, the conclusions and themes of this research may be transferable to other parts of the United Kingdom, although nuances of different services designs might affect the outcomes. In the first review of medication types prescribed by optometrists, the most prescribed medications were topical steroids, dry eye treatments and topical antibiotics, reflecting the high proportion of anterior eye conditions that presented in practice. When developing future services to make use of the skills of optometrists and independent prescribers, commissioners should be sure to consider allowance for on‐going follow up as an important part of the service due to the on‐going nature of conditions that can be managed.

## AUTHOR CONTRIBUTIONS

**Paul Cottrell:** Conceptualization (equal); formal analysis (lead); investigation (lead); methodology (equal); project administration (equal); writing – original draft (lead); writing – review and editing (equal). **Rachel North:** Conceptualization (equal); formal analysis (supporting); methodology (equal); supervision (equal); writing – original draft (supporting); writing – review and editing (equal). **Nicholas John Sheen:** Conceptualization (equal); formal analysis (supporting); methodology (equal); supervision (equal); writing – original draft (supporting); writing – review and editing (equal). **Barbara Ryan:** Conceptualization (equal); formal analysis (supporting); methodology (equal); supervision (lead); writing – original draft (supporting); writing – review and editing (equal).

## CONFLICT OF INTEREST

No authors report any conflict of interest.

## APPENDIX 1 Study survey design

**Table:**

Question		Answer format	Options
1	Date of patient episode	Date	N/A
2	Practice	Selection list	All open practices in the health board area
2.a	Other practice, please provide details	Single‐line free text	N/A
3	Patient age	Selection list	Under 10 11–20 21–30 31–40 41–50 51–60 61–70 71–80 81–90 91–100 101–110
4	Patient sex	Multiple choice	Male Female Other
5	Referred by	Multiple choice	Patient presentation General Medical Practitioner (GP) Pharmacist Optometrist Ophthalmologist Other HES professional Other
5.a	If you selected other, please specify	Multi‐line free text	N/A
6	Form of consultation	Multiple choice	Telephone with patient/carer/guardian Telephone with professional Video with patient/carer/guardian Practice visit Home visit Other
6.a	Reasons for telephone/video only (tick all that apply)	Multiple answer	Patient or carer reluctance Vulnerable group Condition did not warrant practice attendance Other
6.b	If you selected other, please specify	Multi‐line free text	N/A
7	Type of consultation (phone or in person)	Multiple choice	EHEW Band 1 acute (on phone or in person) EHEW Band 3 follow up (on phone or in person) GOS repair (on phone or in person) GOS other (on phone or in person) Other
7.a	If you selected other, please specify	Multi‐line free text	N/A
7.b	Symptoms (tick all that apply)	Multiple answer	None Acute vision problem Chronic vision problem Red eye Flashes Floaters Eye pain/discomfort Headaches Diplopia Other
7.b.i	Findings	Multiple answer	No clinical abnormality Anterior segment Retinal ONH/Visual pathway Binocular vision Post‐operative complications Other
7.b.i.a	Anterior segment	Multiple answer	Dry eye/MGD Eyelid/Eyelash/Lacrimal/Orbit Foreign Body/Other trauma Conjunctiva Cornea/Sclera Cataract/Lens/IOL/PCO Iris/Ciliary Body
7.b.i.b	Retinal	Multiple answer	Dry AMD Wet AMD Other macula Retinal break/detachment Other retinal PVD/other vitreous
7.b.i.c	ONH/visual pathway	Multiple answer	Suspect glaucoma/OHT Optic nerve/Visual pathway/Migraine
7.b.i.d	Binocular vision	Multiple answer	OMB/ refractive error adults OMB/ refractive error children
7.b.i.e	Tentative diagnosis	Single‐line free text	N/A
8	I will take the following action(s) (tick all that apply)	Multiple answer	Advice/regular review Follow‐up Band 3 Foreign Body/Eyelash removal Spectacle Prescription Issued Spectacles made up Spectacle repair Low Vision Aid repair/replacement Referral GP to prescribe Referral Ophthalmologist to prescribe Medication prescription issued Referral to pharmacist for medication Sell/give medication Referral GP other Referral HES Urgent/Emergency Referral other professional Referral HES Advice Report GP Other
8.a	If you selected other, please specify	Multi‐line free text	N/A
8.b	Referral to Pharmacy/advised medication	Multiple answer	Chloramphenicol Dry Eye Drops Anti‐allergy Other Drug
8.c	Referral for medication prescribed/medication prescribed	Multiple answer	Ketorolac trometamol drops Acyclovir tablet Azithromycin drops Dexamethasone drops Chloramphenicol Ointment Acetazolamide tablet Doxycycline hyclate capsule Ofloxacin drops Fluorometholone drops Gentamicin sulphate drops Ketotifen fumarate drops Dexamethasone ointment Monopost/Latanoprost drops Cyclopentolate drops Neomycin ointment Chloramphenicol drops Prednisolone Acetate drops Polymyxin B ointment Hydrocortisone drops Timolol maleate drops Ganciclovir gel Dry eye treatment Olopatadine drops Other
8.c.i	If you selected other, please specify	Multi‐line free text	N/A
9	Additional notes	Multi‐line free text	N/A
